# Histopathology-based breast cancer prediction using deep learning methods for healthcare applications

**DOI:** 10.3389/fonc.2024.1300997

**Published:** 2024-06-04

**Authors:** Prabhu Ramamoorthy, Buchi Reddy Ramakantha Reddy, S. S. Askar, Mohamed Abouhawwash

**Affiliations:** ^1^ Department of Electronics and Communication Engineering, Gnanamani College of Technology, Namakkal, India; ^2^ Department of Computer Science and Engineering, Sri Venkateswara College of Engineering, Tirupathi, India; ^3^ Department of Statistics and Operations Research, College of Science, King Saud University, Riyadh, Saudi Arabia; ^4^ Department of Mathematics, Faculty of Science, Mansoura University, Mansoura, Egypt

**Keywords:** breast cancer, histopathology, Inception V3, Resnet-50, super-resolution generative adversarial networks, transductive long short-term memory

## Abstract

Breast cancer (BC) is the leading cause of female cancer mortality and is a type of cancer that is a major threat to women's health. Deep learning methods have been used extensively in many medical domains recently, especially in detection and classification applications. Studying histological images for the automatic diagnosis of BC is important for patients and their prognosis. Owing to the complication and variety of histology images, manual examination can be difficult and susceptible to errors and thus needs the services of experienced pathologists. Therefore, publicly accessible datasets called BreakHis and invasive ductal carcinoma (IDC) are used in this study to analyze histopathological images of BC. Next, using super-resolution generative adversarial networks (SRGANs), which create high-resolution images from low-quality images, the gathered images from BreakHis and IDC are pre-processed to provide useful results in the prediction stage. The components of conventional generative adversarial network (GAN) loss functions and effective sub-pixel nets were combined to create the concept of SRGAN. Next, the high-quality images are sent to the data augmentation stage, where new data points are created by making small adjustments to the dataset using rotation, random cropping, mirroring, and color-shifting. Next, patch-based feature extraction using Inception V3 and Resnet-50 (PFE-INC-RES) is employed to extract the features from the augmentation. After the features have been extracted, the next step involves processing them and applying transductive long short-term memory (TLSTM) to improve classification accuracy by decreasing the number of false positives. The results of suggested PFE-INC-RES is evaluated using existing methods on the BreakHis dataset, with respect to accuracy (99.84%), specificity (99.71%), sensitivity (99.78%), and F1-score (99.80%), while the suggested PFE-INC-RES performed better in the IDC dataset based on F1-score (99.08%), accuracy (99.79%), specificity (98.97%), and sensitivity (99.17%).

## 1 Introduction

Breast cancer (BC) is the primary cause of death from cancer for women globally. For BC, classification, histopathology, imaging [ultrasound, magnetic resonance imaging (MRI), and computed tomography (CT)], and clinical findings are employed (1). By generating histological tissue for microscopy, pathologists use histology to evaluate the development of cancer. The tissues surrounding cells and structures are represented in histopathological specimens in a variety of ways (2). Hematoxylin and eosin (H&E) is a commonly used histological dye. Cell nuclei are stained blue, and the cytoplasm is stained pink. When stained with H&E, cancer cells commonly have an abnormal appearance, and the pathologist can identify them from normal cells by examining the structure of cells (3, 4). Cancer cells multiply at a rapid pace, and if not correctly identified, they become a serious threat to the patient (5). Early and accurate diagnoses are considered to be significant in improving cancer survival chances (6). Early BC has a persistence rate of approximately 80%, while late-stage illness has a rate of under 20% (7). Among the several diagnostic screening methods for predicting early BC, mammography has become the most expensive and most medically acceptable method (8).

Invasive ductal carcinoma (IDC) and ductal carcinoma *in situ* (DCIS) are two kinds of BC. Only 2% of patients with BC have DCIS, which is a relatively low rate. Additionally, IDC is harmful because it contains whole breast cells. Eighty percent of patients with BC have IDC, with a mortality rate of 10% (9). Histopathological evaluation of breast tissue biopsy images plays a substantial part in the detection of BC (10). Pathological images not only include pathological characteristics of growth, tumor form, and distribution but also provide radiomics benefits such as low cost, high speed, and non-invasiveness (11). Larger size patches that are sampled from a histology image have enough data to be assigned to the patches using the image label (12). However, it is possible that cell-level patches are taken from high-resolution histology images that do not have enough diagnostic information (13, 14). A deep learning approach based on CNN and a clustering model are used to automatically screen more discriminative patches (15). The primary goal is to provide a thorough and efficient method for the multi-classification of histology BC images to enhance diagnostic capabilities, taking into account the aforementioned two factors.

The main contributions are specified as follows:

- Primarily, this research analysis is performed on histopathological images (HIs) of BC using BreakHis and IDC datasets. Previously, the collected datasets are pre-processed by means of super-resolution generative adversarial networks (SRGANs), which belong to advanced deep neural network (DNN) processes and are proficient in producing high-resolution images. SRGANs upsample a low-resolution image into a higher-resolution one with negligible data error.
- Afterwards, high-quality images are processed using data augmentation techniques such as rotation, random cropping, mirroring, and color-shifting to enhance the downstream performance. Data augmentation is vital for many applications, as accuracy increases with the amount of training data. In fact, research studies have found that augmentation significantly enhances the accuracy on image tasks, for example, classification.
- Then, feature extraction is performed by patch-based feature extraction using Inception V3 and Resnet-50 (PFE-INC-RES) to regularize the network's output and diminish the percentage of errors.
- Once the feature extraction is done, a transductive long short-term memory (TLSTM)-based classifier is introduced that efficiently classifies the BC as malignant and benign, which results in higher classification accuracy.

The structure of this manuscript is prepared as follows: Section 2 describes the existing works and Section 3 describes the proposed methodology of this study. Section 4 demonstrates the results and comparison. Section 5 delivers the discussion part. The conclusion is described in Section 6.

## 2 Literature review

Saini and Susan (16) implemented a deep transfer network, VGGIN-NET, to discuss the class imbalance problem in BC. The appropriate layers from the VGG16 were combined with the naïve inception module, dense layer, batch normalization, and flattening to construct the VGGIN-NET architecture. Regularization was employed in the form of data augmentation and dropout to enhance the performance of the VGGIN-NET. Both the majority and minority classes of the VGGIN-NET were effectively classified. Furthermore, the suggested VGGIN-Net is designed to deal with the imbalanced BC dataset and helps to improve the robustness and generalizability of the approach. Whenever the number of layers in VGGIN-Net increases, the convolutional kernel reduces the number of parameters in the convolutional layer, which was equivalent to increasing the regularization. Still, other deep network architectures require the multi-class unbalanced biological dataset for the classification of BC.

Joseph et al. (17) presented a handcrafted feature extraction method and DNN for the multi-classification of BC employing histopathology images on BreakHis. The features were utilized to train DNN classifiers by SoftMax and four dense layers. The presented model avoids overfitting issues by employing the data augmentation method. However, the extraction of several handcrafted features leads to high-dimensional feature vectors, which enhance computing complexity and contain unnecessary or redundant data.

Hamza et al. (18) introduced an improved bald eagle search optimization with synergic deep learning for BC detection employing histopathological images (IBESSDL-BCHI). The introduced method's purpose was to detect and classify BC utilizing HIs. The IBES enables the accurate classification of HIs into two categories: malignant and benign. The IBESSDL-BCHI achieves an improved general efficacy score for the classification of BC. However, to ensure robustness and scalability, the IBESSDL-BCHI method needs to be validated on large-scale real-time datasets.

Khan et al. (19) implemented a MultiNet framework that relies on transfer learning to categorize binary and multiclass BC using the datasets of BreakHis and ICIAR-2018. In the MultiNet framework, three well-known pre-trained models, VGG16, DenseNet-201, and NasNetMobile, were used to extract features from the images of the microscope. A robust hybrid model was created by transferring the extracted features into the concatenate layer. In BreakHis and ICIAR-2018, the MultiNet framework effectively classifies all images as benign. However, establishing a learning rate in the MultiNet framework was difficult since high learning rates lead to unwanted behavior.

Guleria et al. (20) presented a variational autoencoder (VAE) based on a convolutional neural network (CNN) for reconstructing the images to enhance the detection of BC. Images processed from histopathology were presented to detect brain cancer. Various CNN configurations with autoencoder variants were used to produce the prediction outcomes of the VAE. The presented method minimizes the amount of time pathologists use to manually examine the report. However, the computational cost for the architecture of training deep VAEs based on CNN was high.

Liu et al. (21) introduced a novel framework AlexNet-BC model for the classification of BC pathology. The suggested method was trained on ImageNet and fine-tuned by the augmentation. An enhanced cross-entropy loss function was used to penalize and generate forecasts appropriate for uniform distributions. AlexNet-BC has high robustness and generalization characteristics that produce benefits for histopathological clinical systems of computer-aided diagnosis. However, the classifier of SoftMax has a significant threat of overfitting when combined with the loss function of cross-entropy.

The implementation of multiscale voting classifiers (MVCs) for BC histology images was presented by Jakub Nalepa et al. (22). The suggested MVC used clinically interpretable features that are taken from histopathology images of BC. Afterwards, the method was utilized to classify a four-class real-world H&E set using the BACH challenge framework. Finally, the statistical tests supported the trials and showed that the provided classifiers offer high-quality classification, are fast to train, and can draw conclusions quickly. This method helps to reduce the number of false negatives; the number of false positives for these classes partially increases, but it was significantly more cost-effective for medical applications.

Kumari and Ghosh (23) offered a transfer learning method based on the deep convolutional neural network (DCNN) to classify BC from the histological images. The transfer learning method has employed three different DCNN architectures as base models: Densenet-201, Xception, and VGG-16. Each test image has been categorized as malignant or benign after the features from each test image was extracted using three pre-trained base models. The presented method has the potential because of the method's high classification accuracy, which helps doctors accurately diagnose BC in patients. However, the presented method was ineffective in classifying breast histopathology images into various stages of BC.

A deep CNN method has been introduced by Bhausaheb and Kashyap (24) to identify and categorize BC. The deep CNN, which depends on optimization, was utilized for classification, while the V-net design was used for segmentation. The weighted variables are effectively and very steadily trained using the optimization method in conjunction with a deep CNN classifier. However, only histopathologic images have been utilized for verifying the recommended approach.

A data exploratory technique (DET) through predictive algorithms has been developed by Rasool et al. (25) to improve the accuracy of BC diagnosis. The distribution and correlation of features, the removal of recursive features, and the optimization of hyper-parameters are the four layers that make up the DET. Four models' preliminary information were acquired, and predictive algorithms like SVM, LR, KNN, and the ensemble model were used to classify the data. The four algorithms' best performances were taken into account, improving classification accuracy, whereas if tested using the WDBCD dataset, the SVM generates an ineffective outcome.

In order to classify BC, Egwom et al. (26) established a machine learning model called linear discriminant analysis–support vector machine (LDA-SVM), which was used to classify BC, and LDA was used to extract features based on the pre-processed images. To improve accuracy, the information was trained using the cross-validation method. However, the lack of a feature selection procedure made classification a little more difficult.

For the categorization of histopathology images, Fan et al. (27) used transfer learning methods (AlexNet) of the SVM classifier and the traditional softmax classifier. To increase accuracy, the fully connected layer was used in conjunction with SVM. Cross-validation on a fourfold scale is used by the softmax-SVM classifier to increase the effectiveness. In order to identify BC in histopathology images, Ahmed et al. (28) employed PMNet, and the classification procedure makes use of a scaled matrix. Applying the dryad test database, the PMNet system for classification has been assessed. However, it is a laborious and difficult task that is dependent on the knowledge of pathologists.

This work offers a thorough analysis of the advancements in the field of pathological imaging research related to BC and offers dependable suggestions for the design of deep models for various applications. The number of BC diagnoses has significantly increased over time, and this increase has been associated with genetic and environmental problems. There are two groups of BC-related lesions: benign (not cancerous) and malignant (cancerous). Subsequently, because it is dependent on temperature and skin lesions, digital infrared imaging is not the ideal tool for diagnosing early BC. To accelerate the cancer detection process, detection methods have been produced based on the histopathology dataset for BC. Still, conventional feature extraction techniques extract a few low-level characteristics from images, and in order to choose meaningful features, previous information is required, which can be substantially influenced by people. Furthermore, there are few sampled cell-level patches that do not have adequate data to balance the image tag. In order to surpass the problems associated with unsuitable classification, this study presented an advanced classification by means of deep learning algorithm.

## 3 Proposed method

**Figure 1:**
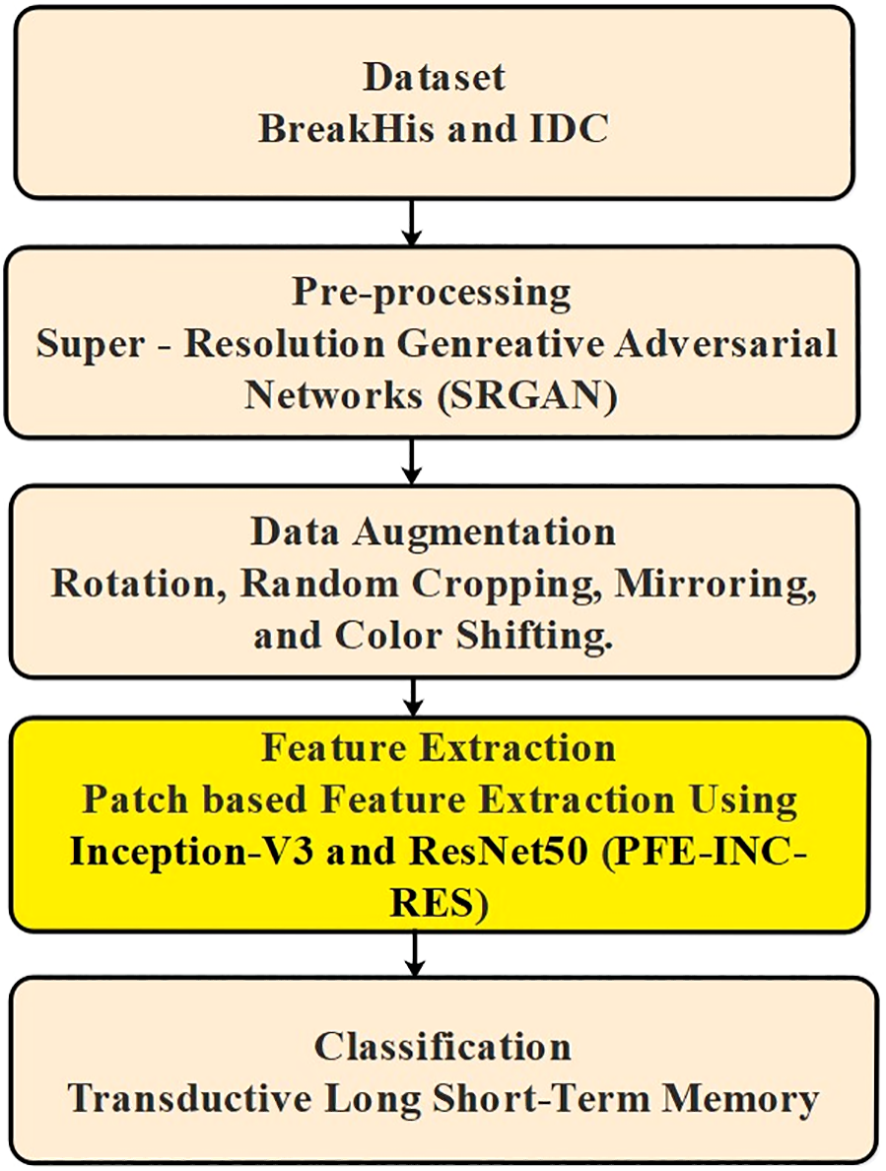
Overall block diagram of the proposed system.

The proposed method included several steps, for example, pre-processing, augmentation, extraction, and classification, where the input data are obtained from BreakHis and IDC. Then, the images gathered from BreakHis and IDC are pre-processed using SRGAN to produce high-resolution images. The pre-processed image then proceeds to the data augmentation stage, where rotation, random cropping, mirroring, and color-shifting are used to either generate new data points or make minor adjustments to the dataset. Next, utilizing PFE-INC-RES, the features from the augmentation are extracted. TLSTM is introduced during the classification stage, where the extracted features are processed, to decrease the frequency of wrong diagnoses and boost classification accuracy. **Figure 1** shows the overall procedure involved in BC classification.

### 3.1 Dataset description

#### 3.1.1 BreakHis dataset

Here, in this dataset, 9,109 microscopic images of breast tumor material obtained from 82 patients with dissimilar magnification factors (40×, 100×, 200×, and 400×) make up BC Histopathological Image Classification (BreakHis). It now has 5,429 malignant samples and 2,480 benign samples. Together with the P&D Laboratory of Pathological Anatomy and Cytopathology in Brazil, the record was originally created (http://www.prevencaoediagnose.com.br). Additionally, researchers might find this dataset to be beneficial because it enables benchmarking and assessment in the future (29). To digitize the images from the breast tissue slides, a Samsung digital color camera SCC-131AN is connected to an Olympus BX-50 system microscope equipped with a relay lens that has a magnification of 3.3× The 1/3" Sony SuperHADTM (Hole-Accumulation Diode) IT (Interline Transfer) CCD (charge-coupled device) used in this camera has a total pixel count of 752×582 with a pixel size of 6.5 µm × 6.25 µm. Magnification factors of 40×, 100×, 200×, and 400×  are used to gather images in three-channel RGB (red–green–blue) TrueColor (24-bit color depth, 8 bits per color channel) color space. The following is how images are acquired at various magnifications: The pathologist initially determines the tumor's identity and establishes a region of interest (ROI). Several images are taken at the lowest magnification  (40×)  to cover the whole ROI. Almost all the time, the pathologist chooses images with only one form of tumor; however, occasionally, images consist of transitional tissue.

**Figure 2:**
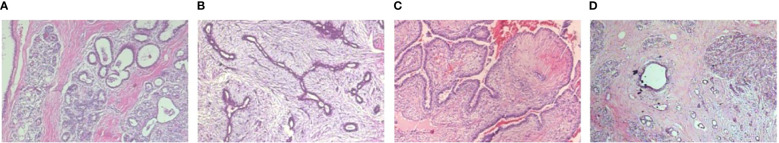
Four types of benign sample images with a magnification of 200× . **(A)** Adenosis, **(B)** Fibroadenoma, **(C)** Phyllodes tumor, **(D)** Tubular adenona.

**Figure 3:**
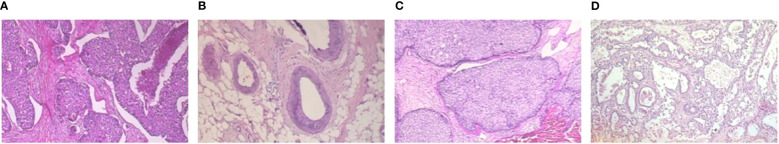
Four types of malignant sample images with a magnification of 200× . **(A)** Ductal carcinoma, **(B)** Lobular carcinoma, **(C)** Mucinous carcinoma, **(D)** Papillary carcinoma.

The latest version's examples were obtained using the surgical open biopsy (SOB), similarly recognized as the partial mastectomy technique. Relative to other needle biopsy techniques, this type of operation extracts a greater quantity of tissue, and it is performed under general anesthesia in a hospital. Depending on how the tumoral cells appear through a microscope, benign and malignant breast tumors can be divided into many kinds. Every image's file name contains information regarding the image itself, including the biopsy technique, the type of tumor, the patient’s identity, and the magnification level. **Figures 2**, **3** display the BreakHis dataset's sample benign and malignant images with a 200× magnification factor. The proposed method is introduced for classifying BC images with a magnification of 200× in benign and malignant tumors. The detected areas have been magnified to 200× for enabling the pathologist to compute cell shape and achieve a higher accuracy.

#### 3.1.2 IDC dataset

**Figure 4:**
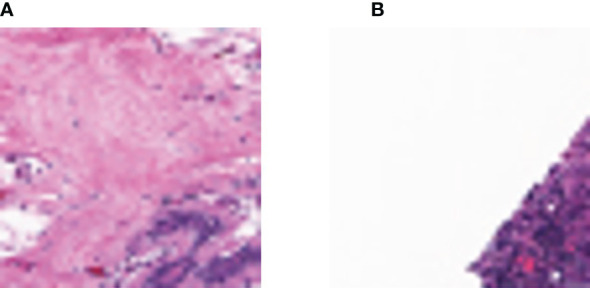
The sample images of the IDC dataset. **(A)** IDC (-), **(B)** IDC (+).

In the Hospital of the University of Pennsylvania (HUP) as well as the Cancer Institute of New Jersey (CINJ), 162 women’s WSIs for diagnosed IDCs have undergone initial digitization. The least prevalent form of BC is IDC. Diagnosticians generally concentrate on zones that contain IDC once the severity of the complete mounted sample is determined (https://www.kaggle.com/datasets/paultimothymooney/breast-histopathology-images). As a result, the common pre-processing stage for automatic aggressiveness grading is to define the precise sections of IDC inside of a whole mount slide. A total of 162 complete mounted slide images of BC samples that had been scanned at 40× made up the original dataset. A total of 277,524 patches of size 50 × 50 were retrieved from it, of which 78,786 were IDC-positive patches and 128,738 were IDC-negative patches. The file name for every patch follows a specific format (30). The image patches were shuffle-selected and categorized into three groups. The training and testing sets have been split as 80:20 from the total dataset. **Figure 4** displays the IDC dataset’s sample images.

After the data collection, the super-resolution reconstruction technique is used to convert the images from low-resolution (LR) to high-resolution (HR) domains, which improves image resolution while restoring the image data. To convert LR images into HR images, traditional image super-resolution algorithms must first pair HR and LR images, then figure out their maps. Since true HR and LR image pairs are hard to come by, the present approach for creating HR-LR image datasets mostly consists of establishing a degradation model, from which the corresponding LR image is derived from the HR image. At the moment, the accuracy of the degradation model has a significant impact on how well super-resolution techniques work with nonideal datasets. To solve the above stated issue, this research suggested a super-resolution GAN model for the collected datasets to enhance the image resolution quality, which is clearly described below.

### 3.2 Preprocessing using super-resolution generative adversarial networks

The components of conventional GAN loss functions and effective sub-pixel nets were combined to motivate the concept of SRGAN. In order to create image details and achieve a better visual impression, SRGAN uses a convolutional neural network as the generation network. This allows the network to be improved by discriminating against training and generating a network. The perceptual loss function is the most notable component of SRGANs. As the generator and discriminator are trained using the GAN design, SRGANs rely on an additional loss function to reach their destination.

In SRGANs, both of the networks are deep convolution neural networks and contain convolution layers and upsampling layers. Each convolution layer is followed by a batch normalization operation and an activation layer. Therefore, in this phase, SRGANs are suggested, which learn the extracted LR images through the multi-scale properties of the two subnetworks, followed by employing high-frequency data across multiple scales to produce HR images. Super resolution (SR) takes the benefits of GAN’s ability to reconstruct an image’s appearance and make an image with high-frequency features (31).

The aim is to generate an SR image from LR using HR’s bicubic procedure. The LR and HR images will be denoted by X  d  Y accordingly. The end-to-end mapping function $G_{\theta }\left(•\right)$ among X  d  Y are solved through the subsequent Equation 1:


(1) $$\hat{\theta }=\begin{array}{c} arg\;min\\ \theta \end{array}\frac{1}{N}\sum_{i=1}^{N}L\left(G_{\theta }\left(X_{i}\right),Y_{i}\right)$$

**Figure 5:**
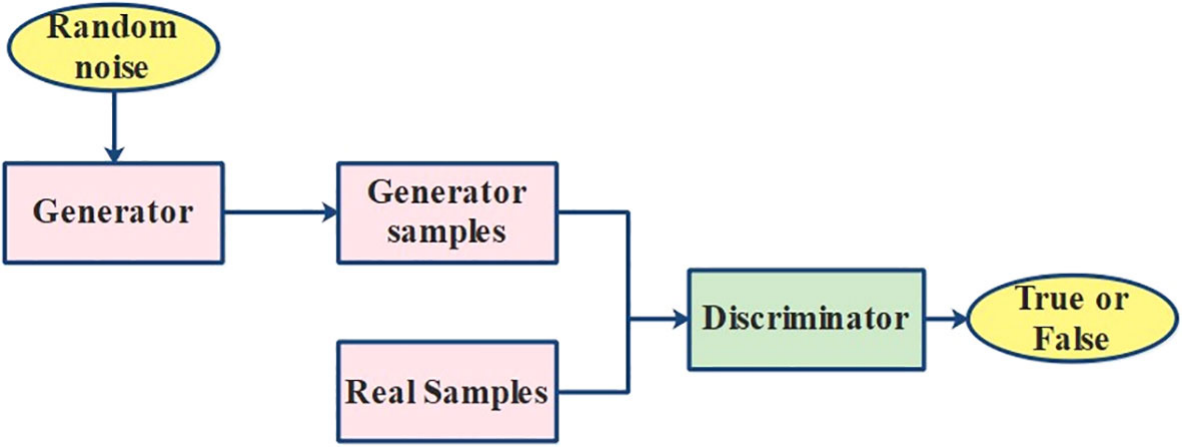
Process of the GAN model.

θ refers to the network parameter set that needs to be enhanced; L(•) refers to the loss function for lessening the variance among X and Y The training sample’s number is referred to as N GAN is a generative framework that contains a generator (*G*) and a discriminator (*D*) as demonstrated in **Figure 5**. Although the discriminator D valuates if the input data are generated by G to be false or real, G receives the data using the initially provided noisy data. Once D is capable of assessing the legitimacy of data and G roduces enough strength to distort D judgment, G and D plays towards one another frequently through this procedure, which continues to return data while enhancing their network features accordingly.

As a result, in order to address the adversarial min–max problem, additionally design a discriminator network $D_{\theta_{D}}$ and optimize it alternately with $G_{\theta_{G}}$ which is defined in Equation 2,


(2) $$\begin{array}{cc} min & max\\ \theta_{G} & \theta_{D} \end{array}E_{Y∼P_{data}\left(Y\right)\left[\log D_{\theta_{D}}\left(Y\right)\right]}+\;E_{X∼P_{data}\left(X\right)}\left[\log\left(1-D_{\theta_{D}}\left(G_{\theta_{G}}\left(X\right)\right)\right)\right]$$

$P_{data}\left(X\right)$ and $P_{data}\left(Y\right)$ signify true sample and generator distribution. Subsequently, the total loss function $L_{total\;}^{SR}$ demarcated as the weighted sum of individual loss functions, which is defined in Equation 3,


(3) $$L_{Total\;}^{SR}=a_{1}L_{MSE}^{SR}+a_{2}L_{Gen\;}^{SR},$$

$L_{Gen\;}^{SR}=\frac{1}{{r2}^{\text{WH}}}\sum_{i=1}^{\text{rW}}\sum_{i=1}^{\text{rH}}(Y_{\text{i,j}}-G_{\theta_{G}}(X){\;}_{\text{i,j}}){\;}^{2}$

$L_{Gen\;}^{SR}=\sum_{n=1}^{N}-\log D_{\theta_{D}}\left(G_{\theta_{G}}\left(X\right)\right)$

Here, the weighting parameters are referred to as $a_{1}\text{ and }a_{2}$ $L_{MSE}^{SR}\;$ signifies the content loss that is exploited as optimization target for SR image; $L_{Gen\;}^{SR}$ signifies the adversarial loss of GAN; r refers to the downsampling factor; W and H represent the image size (32).

### 3.3 Data augmentation

The process of including slightly altered copies of current data without effectively gathering new data from previous training data is known as data augmentation. Additionally, the photographs show variation in elements like magnification, perspective, region of interest, and light, which complicates the classification work. Because of this unpredictability, the classifier may possibly overfit or underfit, which would result in subpar accuracy. The classifier will therefore not generalize very well. The training dataset size can be intentionally increased via data warping or oversampling, which additionally helps the model avoid overfitting by preventing it from the source of the issue. We added to our data using several augmentation settings, including rotation, random cropping, mirroring, and color-shifting (33), to address this overfitting.

#### 3.3.1 Rotation

The key benefit of rotation over flipping is that, when used to some degree, including in the range [−45, +45], it does not completely alter the meaning of the data. The additional benefit when performing rotation at random throughout training is that the algorithm never encounters the same image repeatedly.

#### 3.3.2 Random cropping

Random cropping is a process that involves randomly selecting a portion of the image to crop, which improves durability over partial occlusions. It is carried out to reduce overfitting and introduce regularization throughout training. It is frequently used during training, which prevents the model from seeing the same image repeatedly.

#### 3.3.3 Mirroring

Mirroring across the vertical axis is perhaps the most straightforward data augmentation technique. By flipping an image, a new image is produced. Mirroring continues to maintain the same class of picture for the majority of computer vision applications. This approach is useful in areas like face recognition and object identification datasets where object orientation is not essential. By rotating or mirroring the image, the model could be learned to recognize an object in any direction.

#### 3.3.4 Color shifting

Color shifting is an additional type of data augmentation that is frequently employed to strengthen the learning algorithm’s resistance to changes in the colors of the relevant images. These data augmentation techniques add new instances to the training set while simultaneously expanding the range of inputs the model encounters and absorbs. The model is encouraged to learn broader patterns as a result of both of these factors, which make it harder for the model to merely memorize mappings. These augmented images are specified as input to the extraction phase, where patch-based automatic feature extraction using pretrained architectures is introduced and explained in detail.

### 3.4 Patch-based feature extraction using pretrained architectures

The framework for the multi-class categorization of BC histology images provides a concise summary of the key processes: To maintain important details and to contain features at the cell and tissue levels, firstly extract two distinct kinds of patches from BC histological images using the PFE-INC-RES method.

#### 3.4.1 Sampling patches

The objective is to divide the histology image into the subsequent two categories: malignant and benign. The data gathered from the images have a significant impact on categorization performance. Subsequently, to depict each complete image, employ breast cell-related characteristics and global tissue features. In order to decide if cells are cancerous, cell-level characteristics, including nuclei data form and fluctuation, and cell organization features, consisting of volume and anatomy, are used. The histology images in the dataset have pixels that are 0.42 mm by 0.42 mm in size, and the cell radius is roughly between 3 and 11 pixels. Therefore, to obtain cell-level characteristics, we extract tiny patches of 128×128  pixels. In addition, the sick tissue may have an unusual shape. The starting tissue division is not the only place where invasive cancer can spread (34). To distinguish among *in situ* and invasive carcinomas, knowledge of tissue architecture is crucial. It is impractical for CNNs to acquire characteristics from a large-sized histological image. Then, extract patches of  512×512 pixels to store data on the global tissue patterns based on image size in the provided dataset. Then, from BC histological pictures, extract patches using a sliding window method. Also extract continuous, non-overlapping patches from histology pictures since the 128×128  pixel patches are modest and concentrate on cell-related properties. Additionally, we take overlapping patches of 512×512  pixels by 50% overlap to obtain data regarding constant tissue shape as well as architecture. The label for each extracted patch matches the associated histological image.

#### 3.4.2 Feature extractor

Two pre-trained models are used for patch-based feature extraction, namely, Inception-V3 and ResNet-50, also known as PFE-INC-RES. The cell shape, texture, tissue architecture, and other aspects of the histopathology images vary. For classification, the representation of complicated features is important. The feature extraction method is labor-intensive, and extracting discriminatory features using it is challenging since it requires extensive expert subject knowledge. CNNs have achieved impressive achievements in a variety of disciplines and can directly extract representative characteristics from histopathology images.

##### 3.4.2.1 Inception-V3

Inception-V3 is selected as a preliminary feature extraction method since it has the capability to extract high-level features with a variety of filter modifications (277), as well as an efficient grouping of several forms of convolution process (35). In this technique, a gain of 28% can be achieved utilizing two ( 3×3 ) convolutions. The total number of parameters discovered for the ( 3×3 ) convolution layer is nine. In contrast, a sequence of two ( 1×3 ) convolution layers followed by a third ( 1×3 ) layer, which results in a total of six constraints, represents a 33% reduction overall. The suggested architecture uses a reduction method to overcome issues with conventional pre-trained models.

The images with the dimensions ( 84×84×3 ) are originally supplied to the suggested model for the extraction task without the secondary module, which produces texture features $Inception_{feature\_texture}$ from the final concatenate layer (mixed10) and utilizes a flattened layer that produced an output of $Y_{Inception}$ as shown in Equations 4, 5, where texture feature is converted into a 1D vector.


(4) $$Inception_{feature\_texture}=\;Inception_{mixed10\_layer\left(I\right)}$$

(5) $$Y_{Inception}=flatten\;\left(Inception_{feature_{texture}}\right)$$

##### 3.4.2.2 ResNet-50

To obtain high-level feature extraction, ResNet-50 is utilized to concentrate on low-level features and employ remaining connections in the architecture (36). After becoming saturated during convergence, the Inception-V3 efficiency somewhat declines. In order to overcome these challenges, this dissertation offers ResNet-50 for the FE procedure. ResNet-50 design has 50 layers in five blocks. The residual function, *F*, for each of these blocks has three convolution layers with the dimensions ( 1×1 ), ( 3×3 ), and 1×1 respectively. The output ( Z ) is derived through averaging its input x and residual function  F as shown in Equation 6. The weight matrix $W_{i}$ of three consecutive layers is updated by F on x The input image I which has the shape ( 84×84×3 ), was used to extract features that are provided to ResNet-50, and the output of conv5_block3_out_layer , $ResNet_{conv5\_block3\_out\_layer}\;$ is employed for final classification. $ResNet_{feature\_texture}\;$ previously converted by means of a flattened layer to produce the 1D vector output of $Y_{ResNet}$ that is demarcated in Equations 7 and 8 correspondingly.


(6) $$Z=F\left(x,W_{i}\right)+x$$

(7) $$ResNet_{feature\_texture}=\;ResNet_{conv5\_block3\_out\_layer\left(I\right)}\;$$

(8) $$Y_{ResNet}=flatten\;(ResNet_{feature\_texture})\;$$

The DNN classifier’s functional design uses the extracted feature to predict a class. The concatenation of the two features is done after taking the features from separate models. As a result, the concatenation layer creates a single 1D hybrid vector called $Y_{Hybrid}$ which includes features based on texture and form, i.e., expressed in Equation 9.


(9) $$Y_{Hybrid}=concatenate\;\left(Y_{Inception},\;Y_{ResNet},\right)$$

#### 3.4.3 Screening patches

This section’s goal is to introduce the PFE-INC-RES-based method for screening discriminative 128 by 128 pixel patches. The PFE-INC-RES is trained using highly discriminative data and extract features with patches that are then retrained using the patches. The PFE-INC-RES features that were extracted are used as input for the TLSTM-based classification stage. In this phase, the patient’s BC is separated into benign and malignant varieties based on its nature, which is briefly detailed in the following subsection.

### 3.5 Classification using transductive long short-term memory

An LSTM is a specific kind of recurrent neural network (RNN) that records interunit relationships across extended distances. The LSTM architecture was chosen because it has integrated nonlinear hidden layers among input and output layers. These layers allow the learning of complicated functions and features for better prediction by underpinning the functional relationships from the incoming data (37). This part (38) covers transductive LSTM (T-LSTM), a limited-edition LSTM model. Additionally, all training data point’s influence on T-LSTM’s recommended factors depends on how similar it is to the test data point of length T As a result, the goal is to enhance performance close to the test point, and the effectiveness of the model in forecasting training samples close to the test point is given more attention (39). This makes it conceivable to claim that the test point is a necessity for all linear models.

- Consider $Z^{\left(\eta \right)}$ as a hidden state, the state space model of T-LSTM is mentioned in Equation 10,

(10) $$\{\begin{array}{c} C_{t,\eta }=f\left(C_{t-1,\eta ,}\;h_{t-1,\;\eta ,}x_{t};\;\omega_{lstm,\eta ,}\;b_{lstm,\;\eta }\right)\\ h_{t,\eta }=\;g\left(h_{t-1,\eta ,}\;c_{t-1,\;\eta ,}x_{t};\;\omega_{lstm,\eta ,}\;b_{lstm,\;\eta }\right) \end{array}$$

**Figure 6:**
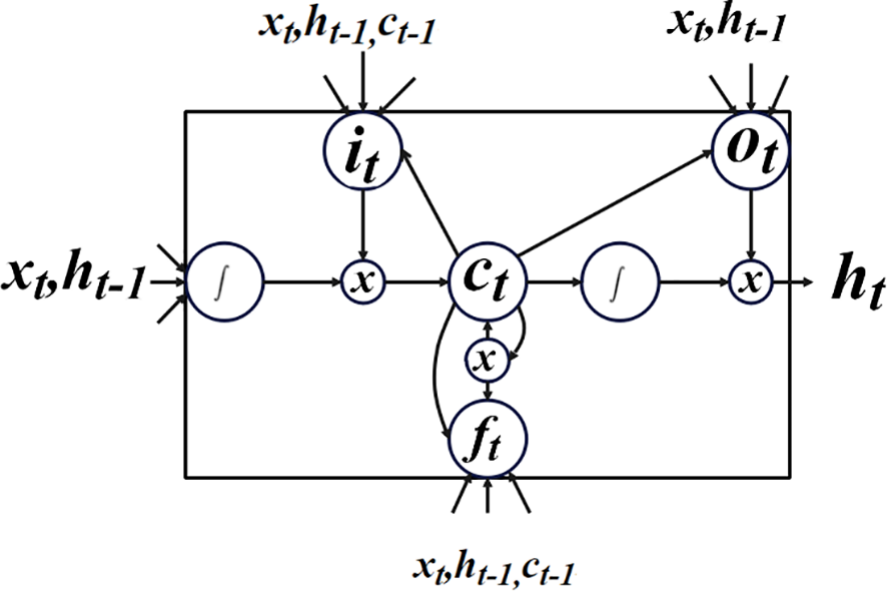
Structure of the T-LSTM process.

It should be noted that the model shown in Equation 14 differs from the previous one in that the model variables in T-LSTM depend on the test point’s feature space, whereas the parameter estimations in LSTM are independent of the test point. The T-LSTM model’s structure is depicted in **Figure 6**.

- Use the subscript η to demonstrate that the linear models depend on the newly supplied data point $Z^{\left(\eta \right)}$ . Note that the test label is considered to be invisible, and that the test point’s function during the training stage is to change the significance of data points, which is close to their feature vectors.
  The prediction is expressed as Equation 11 by utilizing the dense layer,
(11) $${\hat{y}}_{\eta }^{\left(t\right)}=\;\omega_{dense,\eta }^{T}h_{t+T-1,\eta }+\;b_{dense,\eta ,}\;t=1,\ldots ,N,$$
- Here, $\omega_{dense,\eta }^{T}\;\in {\mathbb{R}}^{n\times 1}$ and $b_{dense,\eta ,}\;\in \;\mathbb{R}\;$ are referred to as weights and bias terms.
- To determine the needs of the fresh hidden point, consider $s_{t,\eta }$ as the resemblance among T  Z(t), Z(η), $\omega_{\eta }$ and $b_{\eta }\;$ by signifying every constraint in ( $\omega_{lstm,\eta ,}\omega_{dense,\;\eta }$ ) and ( $b_{lstm,\eta ,}b_{dense,\;\eta }$ ) correspondingly. The objective function is expressed in Equation 12,
(12) $$\left({\hat{\omega }}_{lstm,\eta ,}{\hat{\omega }}_{dense,\eta ,}{\hat{b}}_{lstm,\eta ,}{\hat{b}}_{dense,\eta ,}\right)=\left({\hat{\omega }}_{\eta ,}{\hat{b}}_{\eta ,}\right)=\text{ arg }min{\;}_{\omega_{\eta ,b_{\eta ,}}}J_{\eta }$$

$J_{n\;}=\frac{1}{N}\sum_{t=1}^{\text{N}}S_{t,n\;}({\hat{y}}_{\eta }^{\left(t\right)}-y^{\text{(t)}}){\;}^{2}+{Ὑ}_{{\;}_{\eta }}\omega_{\eta }^{T}\omega_{\eta }$

  where $S_{t,\eta }\;\in {\mathbb{R}}^{+}$ .

${ϒ}_{\eta }\;$ referred to as a tuning parameter in Equation 12. Moreover, the transductive method’s tuning parameter is the number of neurons displayed in LSTM. Additionally, the model in this instance is unable to employ training data from the majority of similar datasets. As a result, there should not be a significant change in the original series pattern, and our study is predicated on that assumption. In feature space, there is not much of a distance between two consecutive data points. Since samples from the final training phase were collected before the test point, they are located in feature space close to the test point. The validation group is chosen to consist of these samples.

- The parameters are dependent on *Z* ^(η)^. Therefore, every unseen subsample has been rehabilitated. It shows every constraint ω_(η)_ and b_(η)_ is diverse for every test point.

Depending on the particulars of the problem, one can question if this approach is suitable for modeling due to the high processing cost of T-LSTM. In contrast to the transductive technique, which trains a different model for each test point, the qualitative methodology’s components are chosen without regard to the test point. When the relationship between components in distinct sections of the input space vary, T-LSTM is also relevant.

- For *Z* ^(η)^ the updated hidden state is described as Equation 13,

(13) $$h_{t^{\prime },\eta }=g\left(\;h_{t^{\prime }-1,\eta ,\;}c_{t^{\prime }-1,\eta ,\;Z_{t^{\prime }}^{\left(\eta \right)};}\;{\hat{\omega }}_{lstm,\eta ,\;}\;{\hat{b}}_{lstm,\eta }\right)$$

- Here, $t^{\prime }$ η…, η+T−1. Later, the final prediction is attained as Equation 14,

(14) $${\hat{y}}_{\eta }^{\left(\eta \right)}=\;{\hat{y}}_{dense,\eta }^{T}\;h_{\eta +T-1,\eta }+{\hat{b}}_{dense,\;\eta }$$

$Z^{\left(\eta \right)}$ is referred to as input. Still, the updated hidden state and prediction in T-LSTM based on the new point $Z^{\left(\eta \right)}$ feature vector, where model constraints are adjusted between training points and $Z^{\left(\eta \right)}$ is designated as the η subscript in $h_{t^{\prime },\eta }$ and ${\hat{y}}_{\eta }^{\left(\eta \right)}$ in Equations 13, 14.

## 4 Results

In this research, two datasets called the BreakHis dataset and IDC are used to evaluate the proposed PFE-INC-RES model. The suggested method is implemented into testing with MATLAB 2021b version 9.11, which has the following system requirements: Windows 11 OS, an Intel Core i7 processor, and 16 GB of RAM. The system performance was estimated by the suggested BC detection approach by means of several metrics. The statistical analysis of accuracy, specificity, precision, sensitivity, F1-score, and its mathematical expression is described in Equations 15–19:


(15) $$Accuracy\;=\;\frac{TP+TN}{TP+TN+FP+FN}\;$$

(16) $$Sensitivity=\;\frac{TP}{TP+FN}$$

(17) $$Specificity=\;\frac{TN}{TN+FP}$$

(18) $$Precision\;=\;\frac{TP}{TP+FP}$$

(19) $$F1-score\;=\;\frac{2TP}{2TP+FP+FN}$$

where TP is true positive, TN is true negative, FP is false positive, and FN is false negative.

### 4.1 Performance analysis on the BreakHis dataset

**Figure 7:**
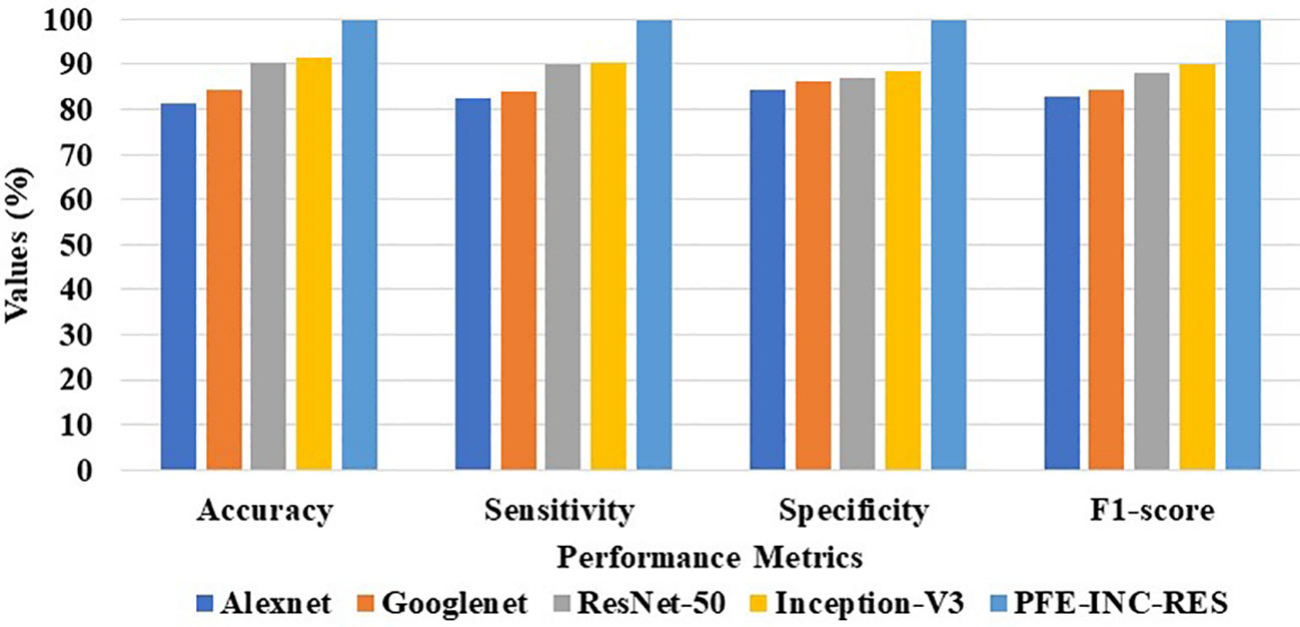
Graphical illustration of feature extraction methods at 40× MF.

The proposed PFE-INC-RES is evaluated on a 40× magnitude factor (MF) in the BreakHis dataset using a variety of extraction methods, which is revealed in **Table 1**; **Figure 7**. This section validates the suggested PFE-INC-RES executed with a training and testing split ratio of 80:20 on 40× using AlexNet, GoogLeNet, ResNet-50, and Inception-V3.

**Table 1:** Analysis of feature extraction methods at 40× MF.

Methods	Accuracy (%)	Sensitivity (%)	Specificity (%)	F1-score (%)
AlexNet	81.37	82.55	84.27	82.88
GoogLeNet	84.41	84.06	86.32	84.34
ResNet-50	90.34	89.87	87.14	88.25
Inception-V3	91.48	90.36	88.37	89.91
PFE-INC-RES	99.84	99.78	99.71	99.80

According to **Table 1**, the proposed PFE-INC-RES clearly exceeds the current classifiers in terms of accuracy (99.84%), sensitivity (99.78%), specificity (99.71%), and F1-score (99.80%). Once the features have been extracted, the TLSTM for the BC classifier on the BreakHis dataset is applied using the PFE-INC-RES technique. Then, using a 40× MF, the TLSTM approach is tested with a variety of classifiers, including MVC, CNN, RNN, deep belief networks (DBNs), and LSTM.

**Figure 8:**
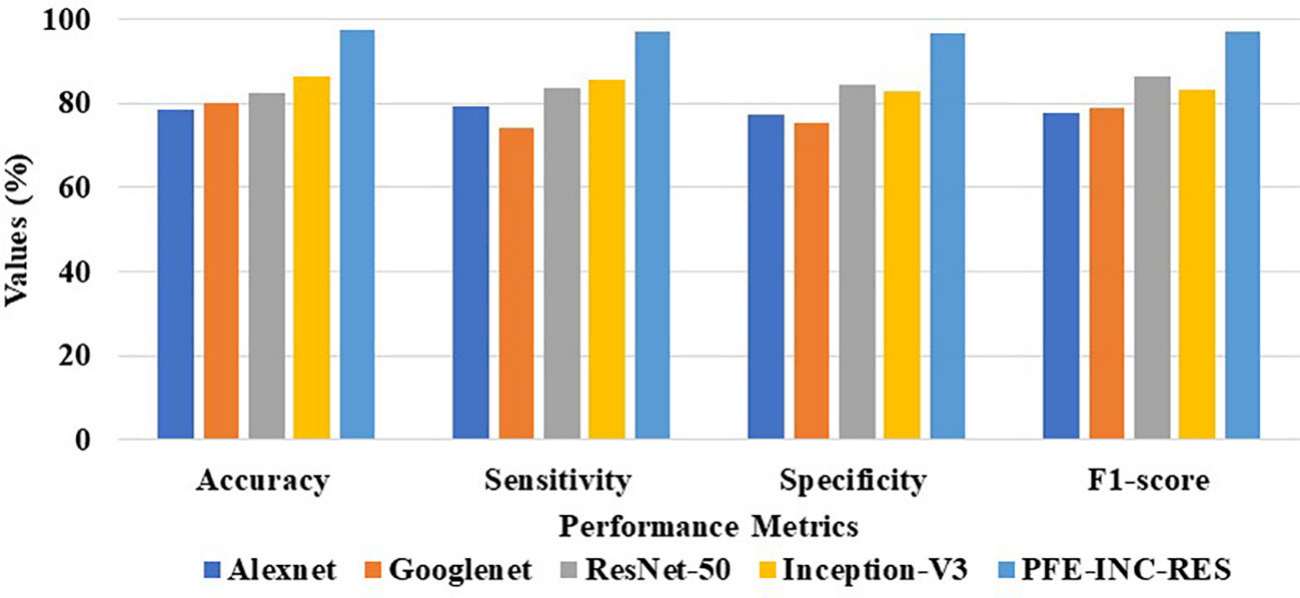
Graphical illustration of feature extraction methods at 100× MF.

**Table 2** clearly demonstrates that the existing LSTM classifier performs classification in terms of accuracy (89.94%), sensitivity (87.49%), specificity (88.65%), and F1-score (88.14%), which are less than those of the TLSTM model. The proposed PFE-INC-RES is then verified using a variety of feature extraction techniques (AlexNet, GoogLeNet, ResNet-50, and Inception-V3), as revealed in **Table 3**; **Figure 8**.

**Table 2:** Analysis of deep learning classifiers on 40× MF.

Methods	Accuracy (%)	Sensitivity (%)	Specificity (%)	F1-score (%)
MVC	81.22	80.13	81.09	80.52
CNN	83.49	82.59	84.68	81.47
RNN	85.39	83.43	86.64	82.34
DBN	88.37	85.58	87.89	86.38
LSTM	89.94	87.49	88.65	88.14
TLSTM	99.84	99.78	99.71	99.80

**Table 3:** Analysis of feature extraction methods at 100× MF.

Methods	Accuracy (%)	Sensitivity (%)	Specificity (%)	F1-score (%)
AlexNet	78.49	79.24	77.16	77.63
GoogLeNet	79.93	74.15	75.39	78.81
ResNet-50	82.34	83.64	84.19	86.19
Inception-V3	86.31	85.57	82.71	83.36
PFE-INC-RES	97.36	97.01	96.48	97.14

According to **Table 3**, the suggested PFE-INC-RES performs better than the currently used feature extraction methods in terms of accuracy (97.36%), sensitivity (97.01%), specificity (96.48%), and F1-score (97.14%) on a scale of 100× MF. Once the characteristics from the PFE-INC-RES technique are extracted, TLSTM is used to classify BC. The TLSTM approach is then tested using a variety of classifiers, including MVC, CNN, RNN, DBN, and LSTM, on a 100× MF, as revealed in **Table 4**.

**Table 4:** Analysis of deep learning classifiers on 100× MF.

Methods	Accuracy (%)	Sensitivity (%)	Specificity (%)	F1-score (%)
MVC	79.49	78.58	76.18	75.47
CNN	81.34	83.46	79.46	79.94
RNN	84.39	84.88	80.34	81.34
DBN	85.17	86.17	85.37	84.41
LSTM	86.62	87.69	84.35	85.16
TLSTM	97.36	97.01	96.48	97.14

**Figure 9:**
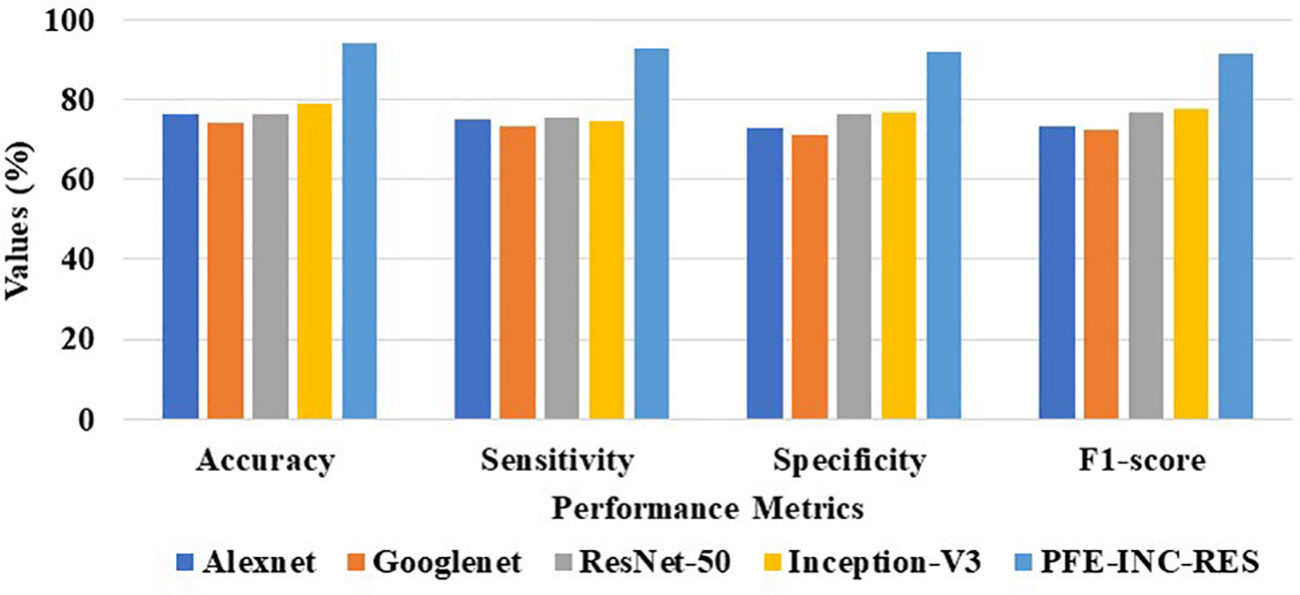
Graphical illustration of feature extraction methods at 200× MF.

**Table 4** clearly proves that the LSTM classifier performs classification in terms of accuracy (86.62%), sensitivity (87.69%), specificity (84.35%), and F1-score (85.16%), which are less than those of the TLSTM model. After that, the proposed PFE-INC-RES is then verified on a 200× MF using a variety of feature extraction techniques, as shown in **Table 5**; **Figure 9**.

**Table 5:** Analysis of feature extraction methods on 200× MF.

Methods	Accuracy (%)	Sensitivity (%)	Specificity (%)	F1-score (%)
AlexNet	76.39	75.16	72.88	73.37
GoogLeNet	74.39	73.18	71.14	72.29
ResNet-50	76.42	75.51	76.18	76.64
Inception-V3	78.84	74.78	76.69	77.79
PFE-INC-RES	94.18	92.86	91.77	91.48

According to **Table 5**, the suggested PFE-INC-RES performs better than the current feature extraction methods in terms of accuracy (94.18%), sensitivity (92.86%), specificity (91.77%), and F1-score (91.48%) by a factor of 20**0×** MF. Once the characteristics from the PFE-INC-RES technique are extracted, TLSTM is used to classify BC. The TLSTM approach is then assessed using a variety of classifiers, including MVC, CNN, RNN, DBN, and LSTM, on a 200× MF, as revealed in **Table 6**.

**Table 6:** Analysis of deep learning classifiers on 200× MF.

Methods	Accuracy (%)	Sensitivity (%)	Specificity (%)	F1-score (%)
MVC	70.29	69.28	68.72	68.21
CNN	73.38	72.24	72.48	71.29
RNN	76.15	75.17	74.39	75.64
DBN	75.64	72.29	72.34	71.59
LSTM	77.49	76.18	75.52	74.48
TLSTM	94.18	92.86	91.77	91.48

**Figure 10:**
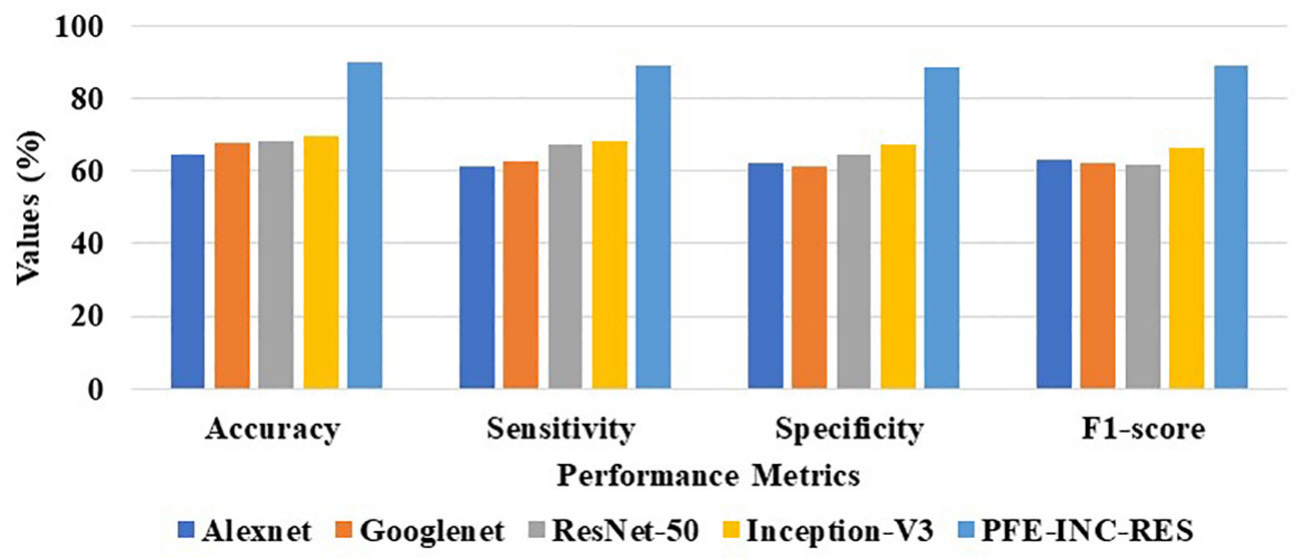
Graphical illustration of feature extraction methods at 400× MF.

**Table 6** clearly demonstrates that the LSTM classifier performs BC classification in terms of accuracy (77.49%), sensitivity (76.18%), specificity (75.52%), and F1-score (74.48%), which are less than those of TLSTM. Next, the proposed PFE-INC-RES is then tested on 400× MF using a variety of extraction methods, as revealed in **Table 7**; **Figure 10**.

**Table 7:** Analysis of feature extraction methods on 400× MF.

Methods	Accuracy (%)	Sensitivity (%)	Specificity (%)	F1-score (%)
AlexNet	64.39	61.37	62.24	63.34
GoogLeNet	67.58	62.73	61.18	62.15
ResNet-50	68.31	67.13	64.43	61.78
Inception-V3	69.49	68.17	67.48	66.51
PFE-INC-RES	90.08	89.18	88.76	89.14

**Table 7** shows that the suggested PFE-INC-RES performs better than the current feature extraction approaches in terms of accuracy (90.08%), sensitivity (89.18%), specificity (88.76%), and F1-score (89.14%) by a factor of 40**0×**. Once the characteristics from the PFE-INC-RES technique are extracted, TLSTM is used to classify BC. The TLSTM approach is then assessed using a variety of classifiers including MVC, CNN, RNN, DBN, and LSTM, on a 400× MF, as shown in **Table 8**.

**Table 8:** Analysis of deep learning classifiers on 400× MF.

Methods	Accuracy (%)	Sensitivity (%)	Specificity (%)	F1-score (%)
MVC	60.49	58.43	59.82	58.66
CNN	63.04	61.82	61.41	62.44
RNN	62.47	61.71	62.08	60.27
DBN	66.38	63.81	64.75	65.17
LSTM	73.36	72.46	71.34	72.45
TLSTM	90.08	89.18	88.76	89.14

**Table 8** clearly demonstrates that the LSTM classifier performs classification in terms of accuracy (73.36%), sensitivity (72.46%), specificity (71.34%), and F1-score (72.45%), which are less than those of the TLSTM model. In terms of BC classification, the suggested technique is more effective than conventional classifiers.

To evaluate the efficacy of the suggested approach, statistical tests such as the Friedman, Wilcoxon, Quade, and Friedman aligned tests have been conducted. In this research, the performances that were utilized to contrast the classification algorithms are investigated using the Friedman test (statistical analysis). The Friedman test is also known as a nonparametric statistical test that is employed in this study. This test assesses the null hypothesis that makes all column effects equal by using the ranks of the data rather than the actual data. In particular, every classifier model was employed in this study to extract features. The chance of reaching the observed sample outcome (*p*-value) in the scalar value within the range [0, 1] is the result of this test. Smaller *p* values generally prevent the null hypothesis from becoming true.

### 4.2 Performance analysis of the IDC dataset

On the IDC dataset for BC classification, the proposed PFE-INC-RES method is evaluated and contrasted with industry-standard feature extraction techniques (AlexNet, GoogLeNet, ResNet-50, and Inception-V3). The TLSTM is then evaluated for BC classification in comparison to common classifiers (MVC, CNN, RNN, DBN, and LSTM). The proposed PFE-INC-RES is evaluated in the IDC dataset using a variety of feature extraction techniques, as shown in **Table 9**. This section validates the suggested PFE-INC-RES on 40× using AlexNet, GoogLeNet, ResNet-50, and Inception-V3.

**Table 9:** Analysis of feature extraction on the IDC dataset.

Methods	Accuracy (%)	Sensitivity (%)	Specificity (%)	F1-score (%)
AlexNet	87.66	84.37	85.41	86.52
GoogLeNet	84.38	86.11	88.19	84.38
ResNet-50	90.27	88.81	87.43	85.56
Inception-V3	91.78	89.48	89.77	88.31
PFE-INC-RES	99.79	99.17	98.97	99.08

**Figure 11:**
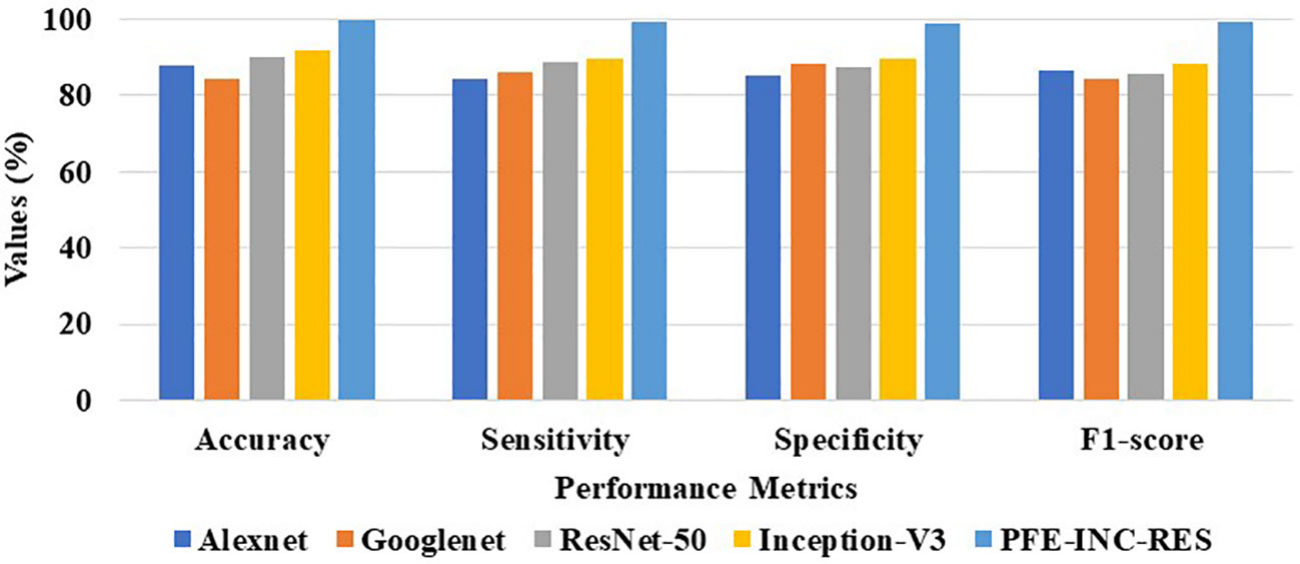
Graphical illustration of feature extraction methods on the IDC dataset.

**Table 9** clearly demonstrates that the proposed PFE-INC-RES operates better than the current extraction methods on the IDC in terms of accuracy (99.79%), sensitivity (99.17%), specificity (98.97%), and F1-score (99.08%), while the existing Inception-V3 has obtained the following values: accuracy (91.78), sensitivity (89.48%), specificity (89.77%), and F1-score (88.31%), which are less than those of the proposed PFE-INC-RES. **Figure 11** shows a graphic representation of feature extraction techniques. Once the characteristics from the PFE-INC-RES technique are extracted, TLSTM is used to classify BC. The TLSTM approach is then tested on the IDC dataset, which is shown in **Table 10**, using a variety of classifiers, including MVC, CNN, RNN, DBN, and LSTM.

**Table 10:** Analysis of existing classifiers on the IDC dataset.

Methods	Accuracy (%)	Sensitivity (%)	Specificity (%)	F1-score (%)
MVC	75.19	74.77	73.58	76.35
CNN	79.46	78.16	79.44	77.48
CNN	79.46	78.16	79.44	77.48
RNN	83.17	81.27	82.75	81.99
DBN	84.88	81.46	80.37	85.28
LSTM	89.34	83.57	84.69	85.43
TLSTM	99.79	99.17	98.97	99.08

**Figure 12:**
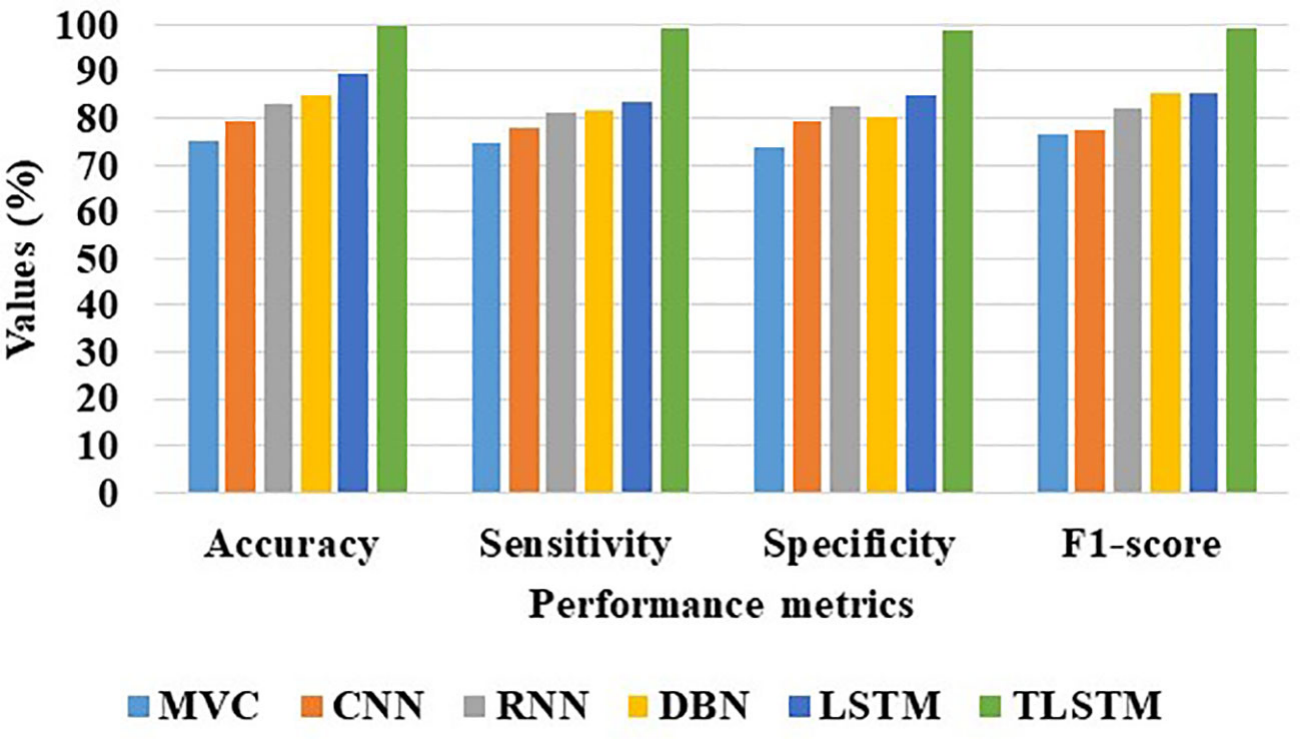
Graphical illustration of various classifiers on the IDC dataset.

**Table 10** clearly demonstrates that the TLSTM classifier performs better than the current classifiers in all performance metrics, while the existing LSTM has obtained the following values: accuracy (89.34%), sensitivity (83.57%), specificity (84.69%), and F1-score (85.43%), which are less than those of the TLSTM classifier. In terms of BC classification, the suggested technique is more effective than conventional classifiers. **Figure 12** shows a graphical representation of classifiers on the IDC dataset.

### 4.3 Comparative analysis

**Figure 13:**
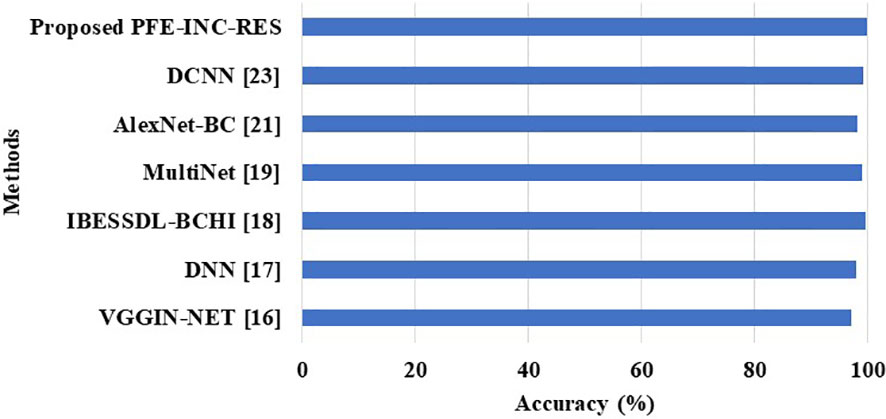
Comparison performances on the BreakHis dataset.

This section provides information about the comparison of the PFE-INC-RES approach to previous studies on BC classification. On the BreakHis dataset, the PFE-INC-RES approach is compared to existing models such as VGGIN-NET (16), DNN (17), IBESSDL-BCHI (18), MultiNet (19), AlexNet-BC (21), and DCNN (23), which is shown in **Figure 13** and **Table 11**. Because of increased exploration and exploitation, which helps in overcoming weak convergence and local optimum conditions, the PFE-INC-RES model performs more efficiently in terms of classification. The PFE-INC-RES model extracts the best characteristics to lessen the issue of overfitting.

**Table 11:** Comparison of the BreakHis dataset.

Methods	Accuracy (%)	Sensitivity (%)	F1-score (%)
VGGIN-NET (16)	97.10	Not Available (N/A)	97
DNN (17)	97.89	98	98
IBESSDL-BCHI (18)	99.63	98.09	98.18
MultiNet (19)	99	99	99
AlexNet-BC (21)	98.15	Not Available (N/A)	Not Available (N/A)
DCNN (23)	99.12	Not Available (N/A)	Not Available (N/A)
Proposed PFE-INC-RES	99.84	99.78	99.80

-, Not Available (N/A).

**Figure 14:**
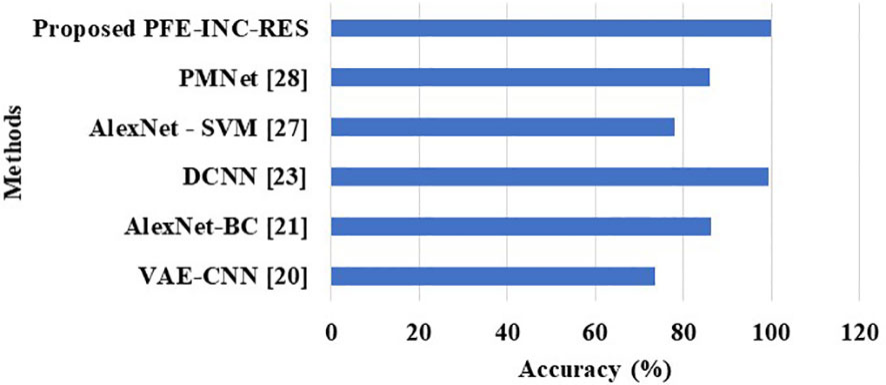
Comparison performance on the IDC dataset.

**Table 11** clearly shows that the existing models such as VGGIN-NET (16), DNN (17), IBESSDL-BCHI (18), MultiNet (19), AlexNet-BC (21), and DCNN (23) have achieved an accuracy of 97.10%, 97.89%, 99.63%, 99%, 98.15%, and 99.12%, respectively, on the BreakHis dataset, while the proposed PFE-INC-RES has obtained a higher accuracy of 99.84%, a sensitivity of 99.78%, and a specificity of 99.80%, which are better than those of the existing models. **Table 12** and **Figure 14** show the evaluation study for BC classification on the IDC dataset.

**Table 12:** Comparison of the IDC dataset.

Methods	Accuracy (%)
VAE-CNN (20)	73.65
AlexNet-BC (21)	86.31
DCNN (23)	99.42
AlexNet-SVM (27)	78
PMNet (28)	86
Proposed PFE-INC-RES	99.79

**Table 12** clearly shows that the PFE-INC-RES model shows a higher performance accuracy of 99.79%, which is better than the existing models, whereas the existing VAE-CNN (20), AlexNet-BC (21), DCNN (23), AlexNet-SVM (27), and PMNet (28) have accomplished an accuracy of 73.65%, 86.31%, 99.42%, 78%, and 86%, respectively, on the IDC dataset. From the overall analysis, the proposed PFE-INC-RES surpasses the conventional models on the basis of all the performance metrics in both BreakHis and IDC datasets.

## 5 Discussion

From the analysis, traditional feature extraction methods only extract a few low-level characteristics from images; prior knowledge is needed to select optimal features. Moreover, a portion of the sampled cell-level patches lack sufficient data to balance the image tag. This work presented a deep learning algorithm-based advanced classification technique to address the problems associated with inappropriate detection and classification. Appropriate data representation is necessary for this research project to succeed. A significant portion of the work is focused on feature engineering, a labor-intensive procedure that utilizes plenty of in-depth domain knowledge to extract valuable features. Additionally, the classification of histology images of BC into benign and malignant categories was the main focus of this study. The overall results from a comparative study show that the suggested PFE-INC-RES achieved better results when associated with existing classifiers. For the BreakHis dataset, the proposed PFE-INC-RES is compared with the existing VGGIN-NET (16), DNN (17), IBESSDL-BCHI (18), MultiNet (19), AlexNet-BC (21), and DCNN (23) classifiers, which achieved an accuracy of 97.10%, 97.89%, 99.63%, 99%, 98.15%, and 99.12%, respectively, on the BreakHis dataset. Similarly, on the IDC dataset, the PFE-INC-RES model shows a higher performance accuracy of 99.79%, which is better than that of the existing models, whereas the existing VAE-CNN (20), AlexNet-BC (21), DCNN (23), AlexNet-SVM (27), and PMNet (28) have accomplished an accuracy of 73.65%, 86.31%, 99.42%, 78%, and 86%, respectively. The suggested PFE-INC-RES performance is evaluated separately for accuracy (99.84%), specificity (99.71%), sensitivity (99.78%), and F1-score (99.80%) based on the overall analysis conducted on the BreakHis dataset. The suggested PFE-INC-RES outperformed the previous models in the IDC dataset in terms of accuracy (99.79%), specificity (98.97%), sensitivity (99.17%), and F1-score (99.08%). The suggested system can be modified for a variety of tasks related to the classification of histological images that are relevant to clinical settings.

## 6 Conclusion

BC is a type of cancer that is associated with a high cancer mortality rate in women. It can take a lot of time to diagnose diseases using HIs when numerous images with varying magnification levels need to be examined. Nowadays, deep learning methods are widely applied in many medical fields, especially those involving classification. However, the learning methods that were in place at the time were consistently producing low classification accuracy. Consequently, deep learning models are employed to analyze histological images of BC using publicly accessible datasets named BreakHis and IDC. The images obtained from BreakHis and IDC are then pre-processed using SRGAN, which creates realistic textures throughout single-image super-resolution. After the image has been pre-processed, it progresses on to the data augmentation stage, where new data points are generated for the dataset by using rotation, random cropping, mirroring, and color-shifting. The features are then extracted from augmentation using PFE-INC-RES. Using feature extractors, the extracted features are chosen from both larger and smaller patches, and the final feature is computed to train a classifier. The TLSTM classifier is included after the extracted features have been analyzed during the classification stage to minimize the count of false diagnoses and increase classification accuracy. The accuracy (99.84%), specificity (99.71%), sensitivity (99.78%), and F1-score (99.80%) of the suggested PFE-INC-RES performances were assessed for the BreakHis dataset, while in the IDC dataset, the proposed PFE-INC-RES achieved better results in terms of accuracy (99.79%), specificity (98.97%), sensitivity (99.17%), and F1-score (99.08%). This analysis clearly states that the proposed PFE-INC-RES significantly outperforms the conventional methods. In the future, an enhanced feature extraction method will be introduced and will be performed using an optimization approach to improve the classification accuracy.

## Data availability statement

The original contributions presented in the study are included in the article/supplementary material. Further inquiries can be directed to the corresponding author.

## Author contributions

PR: Conceptualization, Data curation, Formal Analysis, Methodology, Resources, Visualization, Writing – original draft. BR: Formal Analysis, Investigation, Methodology, Resources, Software, Validation, Writing – original draft. SA: Formal Analysis, Funding acquisition, Methodology, Resources, Software, Validation, Writing – review & editing. MA: Conceptualization, Data curation, Project administration, Resources, Software, Supervision, Validation, Visualization, Writing – original draft, Writing – review & editing.
